# Rational Design of Layered Oxide Materials for Batteries

**DOI:** 10.1021/acs.accounts.5c00074

**Published:** 2025-05-20

**Authors:** Qidi Wang, Chenglong Zhao, Marnix Wagemaker

**Affiliations:** ∥ Department of Mechanical and Energy Engineering, Southern University of Science and Technology, Shenzhen 518055, China; ‡ Shenzhen Key Lab of Energy Materials for Carbon Neutrality, Shenzhen Institute of Advanced Technology, Chinese Academy of Sciences, Shenzhen 518055, China; § Department of Radiation Science and Technology, 378606 Delft University of Technology, Mekelweg 15, 2629 JB Delft, The Netherlands

## Abstract

Layered transition metal (TM) compounds are pivotal in the development of rechargeable battery technologies for efficient energy storage. The history of these materials dates back to the 1970s, when the concept of intercalation chemistry was introduced into the battery. This process involves the insertion of alkali-metal ions between the layers of a host material (e.g., TiS_2_) without causing significant structural disruption. This breakthrough laid the foundation for Li-ion batteries, with materials like LiCoO_2_ becoming key to their commercial success, thanks to their high energy density and good stability. However, despite these advantages, challenges remain in the broader application of these materials in batteries. Issues such as lattice strain, cation migration, and structural collapse result in rapid capacity degradation and a reduction in battery lifespan. Moreover, the performance of batteries is often constrained by the properties of the available materials, particularly in layered oxide materials. This has driven the exploration of materials with diverse compositions. The relationship between composition and structural chemistry is crucial for determining reversible capacity, redox activity, and phase transitions, yet predicting this remains a significant challenge, especially for complex compositions.

In this Account, we outline our efforts to explore rational principles for optimal battery materials that offer a higher performance. The core of this is the concept of ionic potential, a parameter that measures the strength of the electrostatic interaction between ions. It is defined as the ratio of an ion’s charge to its ionic radius, offering a quantitative way to evaluate interactions between cations and anions in crystal structures. By building on this concept, we introduce the cationic potential, which is emerging as a crystallographic tool that captures critical interactions within layered oxide materials. This approach provides insights into structural organization, enabling the prediction of P2- and O3-type stacking arrangements in layered oxides. A key advantage of using the cationic potential is its ability to guide the rational design of electrode materials with improved performance. For example, introducing P-type structural motifs into the material framework can significantly enhance ion mobility, mitigating detrimental phase transitions that often compromise battery efficiency and longevity. Furthermore, ionic potential serves as a representative parameter to quantitatively describe the properties of various TM compositions, providing a straightforward calculation method for designing multielement systems. We anticipate that this Account will provide fundamental insights and contribute to significant advancements in the design of layered materials, not only for battery applications but also for broader fields that require control of the material properties.

## Graphical abstract

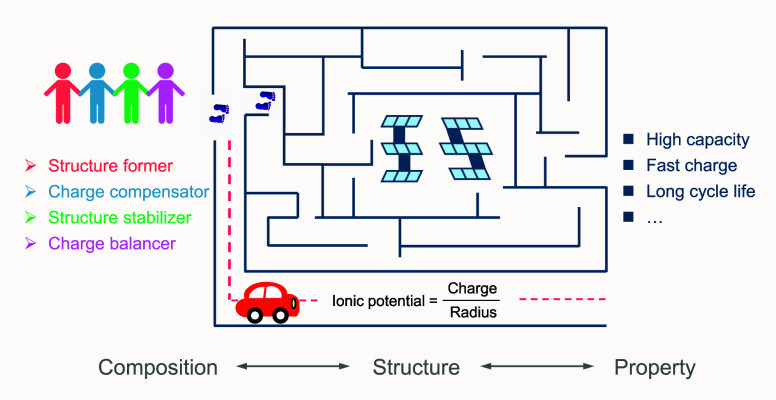

## Key References

-

Zhao, C. ; Wang, Q. ; Yao, Z. ; Wang, J. ; Sánchez-Lengeling, B. ; Ding, F. ; Qi, X. ; Lu, Y. ; Bai, X. ; Li, B. ; Li, H. ; Aspuru-Guzik, A. ; Huang, X. ; Delmas, C. ; Wagemaker, M. ; Chen, L. ; Hu, Y.-S. Rational design of layered oxide materials for sodium-ion batteries. Science 2020, 370 (6517), 708–711 33154140 10.1126/science.aay9972.^1^ *Cationic potential methodology is demonstrated from the ionic potential to determine whether alkali-metal ions in the interlayers remain octahedral rather than switching over to trigonal-prismatic coordination, motivating the exploration of high compositional diversity with enhanced electrochemical performance.*
-

Wang, Q. ; Zhou, D. ; Zhao, C. ; Wang, J. ; Guo, H. ; Wang, L. ; Yao, Z. ; Wong, D. ; Schuck, G. ; Bai, X. ; Lu, J. ; Wagemaker, M. Fast-charge high-voltage layered cathodes for sodium-ion batteries. Nat. Sustain. 2024, 7 (3), 338–347 .^2^ *A principle for the design of fast-charging Na-ion layered electrodes is investigated using the cationic potential method, showing that the introduction of characteristic P3 structural motifs into an O3-type framework serves to promote ionic diffusivity and address phase transitions.*
-

Zhao, C. ; Yao, Z. ; Wang, Q. ; Li, H. ; Wang, J. ; Liu, M. ; Ganapathy, S. ; Lu, Y. ; Cabana, J. ; Li, B. ; Bai, X. ; Aspuru-Guzik, A. ; Wagemaker, M. ; Chen, L. ; Hu, Y.-S. Revealing high Na-content P2-type layered oxides as advanced sodium-ion cathodes. J. Am. Chem. Soc. 2020, 142 (12), 5742–5750 32118416 10.1021/jacs.9b13572PMC7252945.^3^ *The maximum Na content in P2-type layered oxides is explored based on the understanding of Na-ion intercalation chemistry, revealing that a high-content Na presence enhances structural stability and facilitates the oxidation of low-valent cations to their higher oxidation states.*
-

Zhao, C. ; Ding, F. ; Lu, Y. ; Chen, L. ; Hu, Y.-S. High-entropy layered oxide cathodes for sodium-ion batteries. Angew. Chem., Int. Ed. 2020, 59 (1), 264–269 10.1002/anie.20191217131621145.^4^ *A multiple-component layered oxide with several transition metals was first demonstrated as a cathode for Na-ion batteries, showing highly reversible phase transition behavior upon cycling due to the accommodation of the local changes on the host structure.*

## 1 Introduction

The integration of intermittent renewable energy sources, such as solar and wind energy, necessitates the development of sustainable electrical energy storage systems for stationary applications. Rechargeable batteries, particularly lithium (Li)-ion batteries, have been extensively used in portable electronic devices and electric vehicles due to their high energy conversion efficiency and flexible energy storage capabilities.^5^ In comparison, sodium (Na)-ion batteries show potential advantages for smart grids and large-scale grid applications due to the abundance and low cost of Na. Both Li-ion and Na-ion batteries operate on a similar rocking-chair mechanism, where ion insertion and extraction from electrode materials enable high reversibility and stability.^6^ Over the past few decades, these technologies have undergone rapid development; however, high-performance electrode materials remain a critical area for further advancement, particularly for large-scale applications.

Exploring new materials with improved chemical and dynamic stability as well as higher energy storage performance has led to the discovery and optimization of various insertion compounds, including oxides, polyanionic compounds, and others.^7^ Among these, layered oxides with the general formula A_*x*_TMO_2_ have been the most significant family of electrode materials for Li-ion batteries since the 1980s, presenting compositional diversity and tunable electrochemical performance through element substitution.^8–10^ Here, A represents an alkali metal such as Li, Na, and K, while TM refers to one or more transition metal elements that facilitate redox reactions associated with ion (de)­intercalation. Additionally, TM ions exhibit diverse electron configurations, which result in a variety of oxidation states. The combination of these oxidation states drives the formation of different layered structures in alkali-metal oxides.

The crystal structures of A_*x*_TMO_2_ compositions, dominated by specific 3d TMs, are summarized in Figure 1a. For Na-ion layered oxides, layered materials with Ti^4+^ include O3-Na_0.8_Ni_0.4_Ti_0.6_O_2_ and P2-Na_0.66_Li_0.22_Ti_0.78_O_2_. For the V^4+^/V^3+^ systems, layered materials include O3-NaVO_2_ and P2-Na_0.71_VO_2_. For Cr^3+^, the reported material is O3-NaCrO_2_. Mn^4+^/Mn^3+^ layered materials include O3-NaMnO_2_, P2-Na_2/3_MnO_2_, and P2-Na_2/3_Ni_1/3_Mn_2/3_O_2_. The Fe^3+^-containing layered material is O3-NaFeO_2_. Co^4+^/Co^3+^/Co^2+^ layered materials include O3-NaCoO_2_, P2-Na_2/3_CoO_2_, and P2-Na_2/3_Co_2/3_Te_1/3_O_2_. For Ni^3+^/Ni^2+^, layered materials include O3-NaNiO_2_, and O3-NaNi_1/2_Mn_1/2_O_2_. Cu^3+^ layered material includes O3-NaCuO_2_. Compared with Li- and K-ion layered oxides, Na-ion layered oxides allow a broader range of element substitutions at the TM sites. Lattice sites of the TMs can also be occupied by inactive elements such as Li, Mg, Al, Ca, Ru, Sn, Sb, Te, Ir, Bi, and even vacancies,^11,12^ which can enhance the stability of the structural framework through alternative bonding interactions. This flexibility in element substitution opens up extensive opportunities for designing and synthesizing new layered oxide materials. Additionally, in Na_*x*_TMO_2_ layered oxides, the average valence state of the elements at the TM sites typically ranges from 3+ to 4+, depending on the Na ion content (*x*). However, elements with valence states of 1+, 2+, 3+, 4+, 5+, or 6+ are often used to substitute for TM elements, resulting in stoichiometric ratios that tend to follow specific patterns (Figure 1b). For example, when the Na content is 2/3, the composition of the TM site can include combinations with metal elements (Me), such as 2/9­(A^+^) and 7/9­(Me^4+^), 1/2­(Me^2+^) and 2/3­(Me^4+^), 5/9­(Me^2+^) and 4/9­(Me^5+^), 2/3­(Me^2+^) and 1/3­(Me^3+^), or 2/3­(Me^3+^) and 1/3­(Me^4+^). Elements with the same valence state can often substitute for one another to some extent, and different elements can achieve charge balance (e.g., 2Me^3+^ = Me^2+^+Me^4+^).

**Figure 1:**
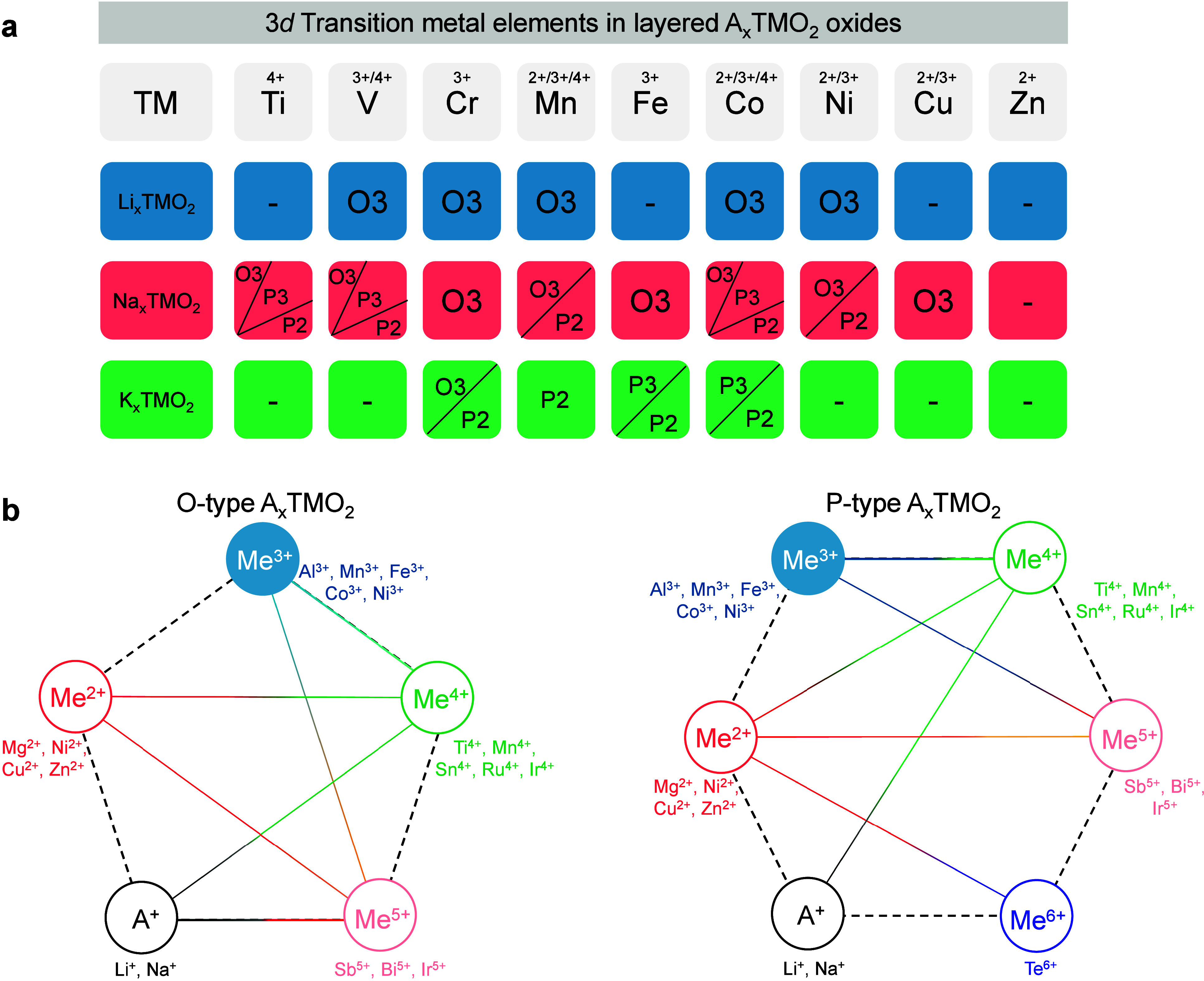
**Structural and compositional diversities of alkali-metal layered oxides**. (a) Redox couples and structures of 3d transition metals (TMs) used in layered electrode materials. These structures represent compositions that have been reported to be directly synthesizable. (b) Element composition of the O- and P-type layered oxide materials.

Upon cycling, the oxidation states of the TM ions change with the insertion and extraction of Na ions. Consequently, the Na content plays a critical role in determining whether a composition adopts an O- or P-type layered structure. For materials that can be directly synthesized, the O-type structures are typically associated with higher Na content, with the Na content in the O3-type structures generally ranging from approximately *x* = 0.8 to 1. In contrast, P-type structures are more commonly observed at lower Na contents, typically within the range of 0.5 < *x* < 0.75. However, it has been observed that the Na content alone does not directly dictate the layered configuration.

In fact, in addition to the Na contents, factors such as the nature of the TM ions and synthesis conditions play a crucial role in determining whether a material adopts an O- or P-type layered structure. As an example, Mn is particularly suitable for developing Na-ion battery technology for large-scale energy storage due to its abundance. Figure 2a summarizes the phase diagrams of Na_*x*_MnO_2_ and Mn-based oxides Na_*x*_Mn_*y*_Me_1–*y*_O_2_ doped with other Me ions, as reported in the literature. In the Na–Mn–O ternary system, as the Na content increases, a structural transition occurs around *x* ≈ 0.44, shifting from three-dimensional tunnel structures to two-dimensional layered structures. Representative compositions include tunnel-structured Na_0.44_MnO_2_, P′2-layered Na_0.52_MnO_2_, P2-layered Na_0.74_MnO_2_, and O3 or O′3-layered NaMnO_2_. However, the redox potential of Mn^4+^/Mn^3+^ is relatively low (∼2.7 V), and the Jahn–Teller effect associated with Mn^3+^ induces lattice distortion, necessitating alternative strategies to enhance the electrochemical performance of Mn-based cathodes.^13^ Elemental doping is a potential approach to address these challenges. Figure 2a illustrates the structural phase diagrams for Na_*x*_Mn_*y*_Me_1–*y*_O_2_ doped with various Me ions, including monovalent ions (e.g., Li^+^), divalent ions (e.g., Mg^2+^, Ni^2+^, Cu^2+^, and Zn^2+^), combinations of monovalent and divalent ions, monovalent and trivalent ions (Fe^3+^ and Co^3+^), and divalent and trivalent ions as well as combinations involving mono-, di-, and trivalent ions. Most of these compositions form P-type structures (including P2 and P3) at a low Na content and transition to O3-phase structures at a high Na content. Although this discussion uses Mn-based materials as examples to explore the influence of Na content on phase structures, the actual processes and structural outcomes are often more complex.

**Figure 2:**
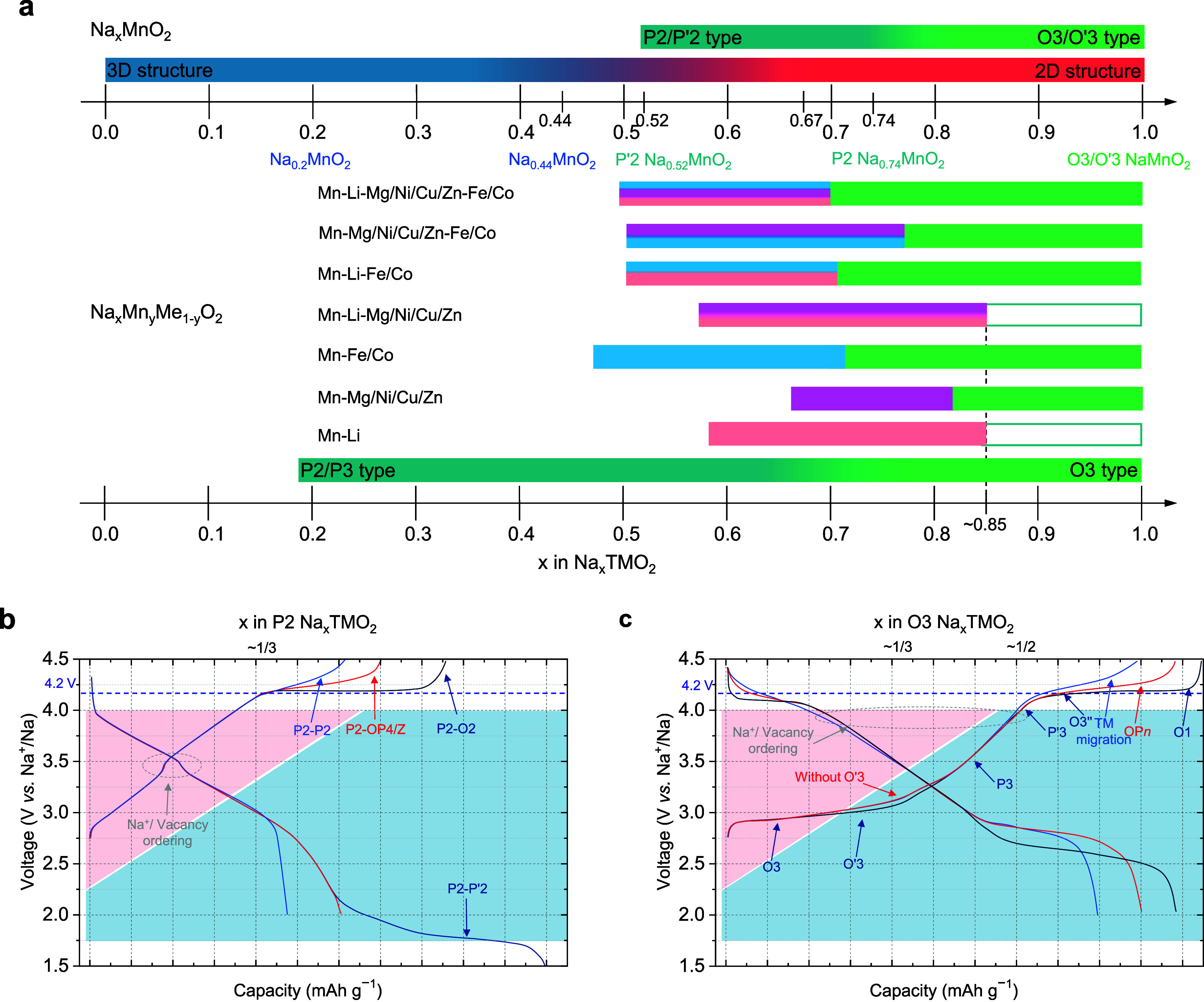
**Structural and compositional evolutions of alkali-metal layered oxides**. (a) Structure composition of Na_*x*_MnO_2_ and Na_*x*_Mn_*y*_Me_1–*y*_O_2_ compounds. (b) Summary of structure evolution for P2-type layered oxide materials during the discharge–charge process. (c) Summary of structure evolution for O3-type layered oxide materials during the discharge–charge process.

On the other hand, electrodes often undergo complex structural transitions upon cycling, which can significantly impact the cycling stability of batteries. Some typical structural transitions observed in P2-type and O3-type layered materials during electrochemical processes are summarized in Figure 2b,c. Figure 2b illustrates the structural evolution behaviors of P2-type materials along with the corresponding schematic charge–discharge curves. Typically, within the voltage range of 2.0–4.0 V, structural transitions involving Na-ion/vacancy ordering or charge ordering may occur.^14,15^ When charged to approximately 4.15 V or higher, the material may undergo one of several structural transitions, such as P2-to-O2 transition, P2-to-OP4/Z transition, or P2 structure solid-solution transition.^16,17^ Upon discharging to around 1.5 V, structural changes, such as a transition from P2 to P′2, can occur due to octahedral distortion.^13^ These transitions reflect the complex interplay of electrochemical and structural dynamics that influences the performance and stability of P2-type materials in Na-ion batteries. Figure 2c summarizes the common structural transitions of Na-ion O3-type layered oxide cathodes. At high voltages, transitions to O1 or OP*n* phase or behaviors involving TM migration seem nearly unavoidable unless the charging cutoff voltage is limited to below approximately 4.0 V.^18–21^ In contrast, structural changes to the O′3, P′3, or O3″ phase at voltages below 4.0 V can be mitigated to some extent through strategies such as element doping. However, the O3-to-P3 structural transition is inherently difficult to suppress. Two approaches can be considered for managing this transition: reducing the proportion of the O3-type phase and increasing the proportion of the O3-type phase.

Layered oxide materials possess diverse chemical compositions, and each composition exhibits distinct electrochemical properties and structural transition characteristics. Gaining a systematic and rational understanding of these underlying features is essential for the design and development of high-performance battery materials. In this Account, we discuss the fundamental scientific issues of layered oxide materials, drawing our insights from nearly a decade of dedicated research. We first introduced the cationic potential method as a predictive tool to understand the stacking structures of these materials, as demonstrated by the successful design and synthesis of high-Na P2-type and low-Na O3-type layered electrodes with enhanced performance. Additionally, we explored a multicomponent approach to identify promising chemical composition spaces for developing materials with tunable functionalities. These findings collectively illuminate the structural origins of layered oxides, particularly for Na-ion batteries, offering valuable guidance for the rational design of advanced materials. Lastly, future directions are proposed to inspire continued progress based on our expertise.

## 2 Cationic Potential Method for Predicting Layered Stacking Structures

Compared to octahedral O-type Li-ion layered oxides, Na-ion oxides exhibit a notable difference: they can adopt both O-type and P-type stacking arrangements, with the latter being characterized by prismatic Na-ion coordination.^22^ These stacking configurations exhibit distinct electrochemical performance, where the most studied layered stacking configurations are the P2 and O3 types (Figure 3a). The P2 type corresponds to ABBA oxygen stacking, while the O3 type follows an ABCABC stacking pattern.^23^ P2-type oxides generally offer higher Na-ion conductivity and better structural stability compared to their O3 counterparts, contributing to better power density and cycling stability.^24^ However, the lower initial Na content of P2-type materials limits their reversible capacity during the first charge compared to the Na-rich O3-type oxides.^25^ In 2018, we found that the crystal structures of P2- and O3-type oxides can be distinguished by the ratio of the interlayer distance in the Na metal layer, *d*_(O–Na–O)_, to that in the TM layer, *d*_(O–TM–O)_, with a ratio of approximately 1.62 serving as a characteristic marker^26^ (Figure 3a). The larger ratio observed in P2-type oxides results from a more localized electron distribution within the TMO_2_ slabs. This leads to weaker repulsion between adjacent NaO_2_ slabs and stronger repulsion between adjacent TMO_2_ slabs. These findings highlight the critical role of the electron distribution in determining the competition between P- and O-type stackings in layered oxides.

**Figure 3:**
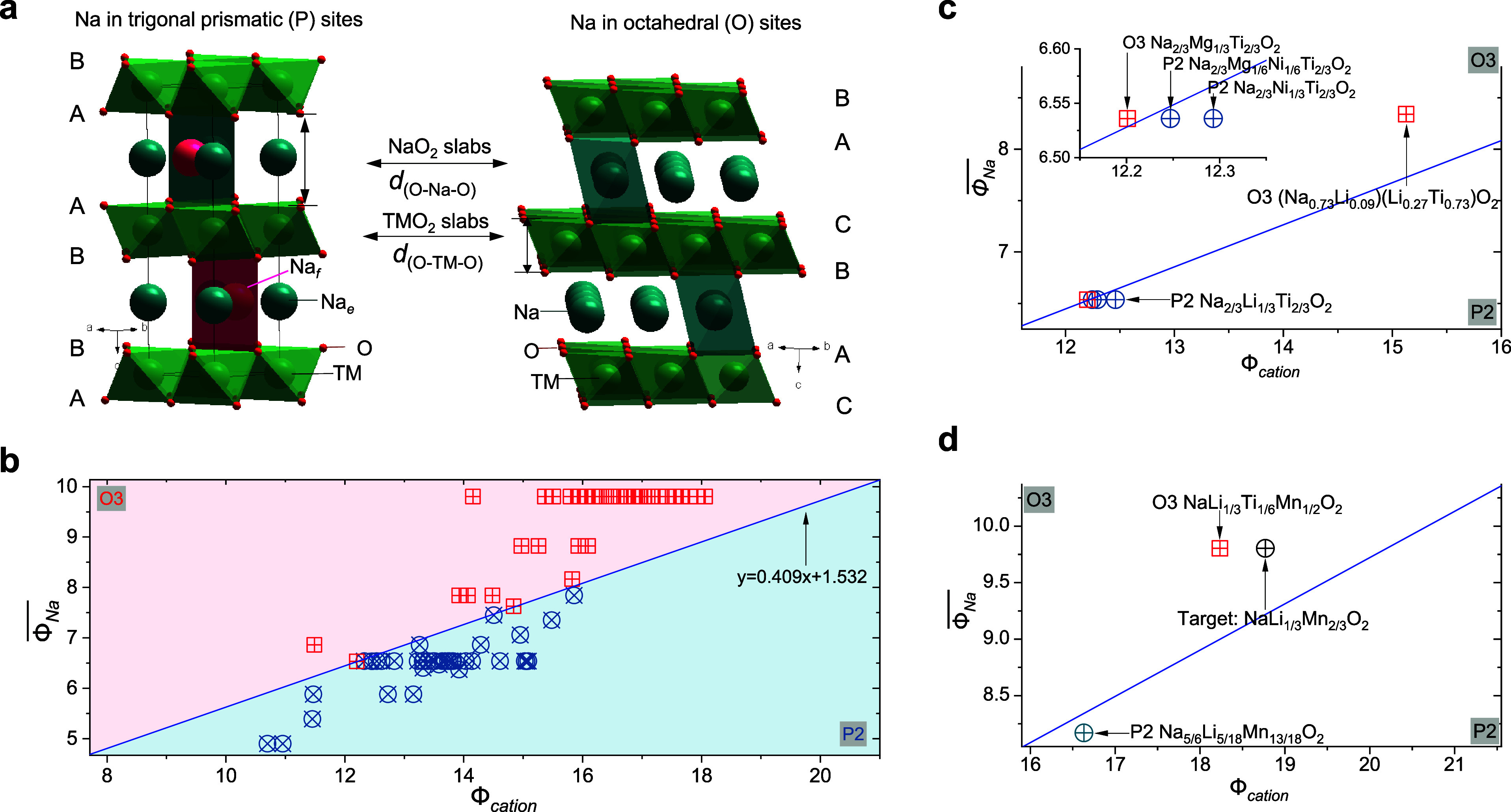
**Crystal structures of layered oxide materials**. (a) Schematic illustration of crystal structures of representative P2-type and O3-type layered oxides. (b) Cationic potentials of representative P2-type and O3-type Na-based layered oxides, considering the different Na contents, oxidation states of TMs, and TM composition. (c) Analysis of cationic potentials on P2- and O3-type oxides. (d) Designing O3- and P2-type oxides based on the proposed cationic potential. Reproduced with permission from ref 1. Copyright 2020 American Association for the Advancement of Science.

The ionic potential (Φ), introduced by G. H. Cartledge,^27^ serves as a measure of the charge density on the surface of an ion. It is defined as the ratio of the charge number (*n*) to the ion radius (*R*), providing insight into the cation’s polarization capability. To develop a descriptor for layered oxides, we defined a new parameter termed the “cationic potential” as the extent of a cation’s electron density and polarizability normalized by the ionic potential of the anion (oxygen), which offers a focused way to quantify these characteristics:

1 $$\Phi_{\mathrm{cation}}=\frac{\overset{®}{\Phi_{\mathrm{TM}}\;}\overset{®}{\Phi_{\mathrm{Na}}\;}}{\overset{®}{\Phi_{O}\;}}$$

where $\overset{®}{\Phi_{\mathrm{TM}}\;}$ represents the weighted average ionic potential of TMs, defined as $\overset{®}{\Phi_{\mathrm{TM}}\;}=\sum \frac{w_{i}n_{i}}{R_{i}}$ , in which *w*_*i*_ is the content of TM_*i*_ having charge number *n*_*i*_ and radius *R*_*i*_, and $\overset{®}{\Phi_{\mathrm{Na}}\;}$ represents the weighted average ionic potential of Na, defined as $\overset{®}{\Phi_{\mathrm{Na}}\;}=\frac{x}{R_{\mathrm{Na}}}$ . Note that charge balance in Na_*x*_TMO_2_ composition demands ∑*w*_*i*_ *n*_*i*_ = 4 – *x*, where *x* represents Na content and 4 is the total oxidation state to charge-compensate for the charge of O^2–^.

By building the relationship between the cationic potential (Φ_cation_) and the average Na ionic potential ( $\overset{®}{\Phi_{\mathrm{Na}}\;}$ ) for reported P2- and O3-type layered oxides, we could visualize the phase map as shown in Figure 3b. This map highlights distinct regions for P2- and O3-type structures, demonstrating that the cationic potential serves as a reliable descriptor for interslab interactions and the structural competition between these two types. A higher cationic potential indicates a greater extension of the TM electron cloud and increased electrostatic repulsion between layers, favoring the formation of the P2-type structure. This structure features more covalent TM–O bonds and a larger *d*_(O–Na–O)_. Conversely, a higher mean Na ionic potential, achieved by increasing the Na content, enhances the shielding effect, reducing electrostatic repulsion between TMO_2_ slabs and promoting the O3-type structure. The phase map reveals that minor changes in TM or Na content can shift the balance between P2- and O3-type phases. For instance, in the P2-Na_2/3_Ni_1/3_Ti_2/3_O_2_ composition,^28^ substituting Ni^2+^ with Mg^2+^, which is feasible due to their similar ionic radii, leads to Na_2/3_Mg_1/3_Ti_2/3_O_2_. The smaller ionic potential of Mg^2+^ compared to Ni^2+^ reduces the cationic potential, decreasing the covalence of Mg/Ti–O bonds. This in turn increases the charge on oxygen atoms and weakens the repulsion between TM layers, favoring the O3-type structure. The cationic potential of this composition predicts the O3-type structure, a result that would be challenging to determine using conventional electrostatic energy calculations. Furthermore, substituting 1/6 Mg^2+^ with Ni^2+^ in Na_2/3_Mg_1/3_Ti_2/3_O_2_ to form Na_2/3_Ni_1/6_Mg_1/6_Ti_2/3_O_2_ shifts the structure back to the P2 type. This example illustrates how compositions near the phase boundary are highly sensitive to small variations in the cationic composition, as shown in Figure 3c.

The primary distinction between P- and O-type structures lies in the electrostatic polarization between the AO_2_ and TMO_2_ slabs. This makes the proposed cationic potential method a valuable tool for differentiating Na-ion layered oxides. Guided by this approach, we have successfully designed and synthesized high-capacity (>200 mAh g^–1^) layered cathodes of both O3-type NaLi_1/3_Ti_1/6_Mn_1/2_O_2_ and P2-type Na_5/6_Li_5/18_Mn_13/18_O_2_ materials starting from NaLi_1/3_Mn_2/3_O_2_. This composition, characterized by a relatively low molecular weight and sufficient charge compensation via oxygen redox chemistry, offers a high theoretical specific capacity. However, it poses significant challenges for preparation through conventional solid-state reactions of NaLi_1/3_Mn_2/3_O_2_.^29^ By carefully adjusting the Na content and/or TM composition, the cationic potential accurately predicted the stacking structures of these two materials (Figure 3d), which were subsequently synthesized in the predicted forms by using the same synthesis method. To date, the cationic potential method has been broadly applied to predict P2- and O3-type layered structures, leading to the discovery of various new materials. Examples include O3-type Na_0.826_Li_0.06_Ni_0.27_Mn_0.5_Fe_0.1_Ti_0.07_O_2_,^30^ O3-type Na_0.75_Ni_0.25_Cu_0.10_Fe_0.05_Mn_0.15_Ti_0.45_O_2_,^31^ O3-type NaNi_0.45_Zn_0.05_Mn_0.4_Ti_0.1_O_2_,^32^ and P2-type Na_0.67_Ni_0.3_Zn_0.03_Mn_0.52_Ti_0.15_O_21_O_2_.^32^ Since most currently known layered materials are either low-Na-content (*x* = 2/3) P2-type oxides or high-Na-content (*x* = 1) O3-type oxides, we propose that further research could focus on the development of high-Na-content P2-type oxides and low-Na-content O3-type oxides. It is important to note that relying solely on the cationic potential approach may not accurately predict phase transitions, as it is based purely on compositional calculations. A comprehensive understanding of material properties requires a consideration of additional factors, highlighting the need for further investigation.

## 3 High-Na-Content P2-Type Layered Cathodes

Generally, P2-type structures enable fast Na^+^ diffusion due to their open prismatic diffusion pathways between TMO_2_ slabs, presenting higher cycle and rate capabilities. However, these electrodes typically exhibit a low initial charge capacity of ∼80 mAh g^–1^ below 4.0 V^33^ or a low average voltage (<3.2 V).^34^ Additionally, they are prone to detrimental phase transitions, such as P2 to O2 or OP4/Z phases upon charging to a higher voltage (Figure 2b), which compromises the cycling stability. To address the structural instability, ion substitution and doping strategies are widely employed.^35^ For example, substituting 5 mol % Mg for Ni in Na_2/3_Ni_1/3_Mn_2/3_O_2_ can partially suppress the global O2 phase transition, though it induces a local OP4/Z phase transition.^36^ On the other hand, to enhance charge capacity beyond 100 mAh g^–1^ below 4.0 V, TM^3+^-based P2-type oxides containing TM ions, such as Mn^3+^,^33^ and Fe^3+^, have been explored. However, these materials generally exhibit lower redox potentials (below 3.0 V), which reduces the overall energy density, and they often experience structural transitions at both high voltage (P2 to O2, OP4/Z) and low voltage (P2 to P′2). Balancing these demands remains challenging, highlighting the need for a deeper fundamental understanding.

As we discussed before, Na content is a critical factor influencing the structural stability of P2-type materials, where during charging the reduction in Na^+^ shielding promotes the sliding of the TMO_2_ slabs, resulting in structural transitions from P-type to O-type stacking. Retaining more Na^+^ within the P2-type structure is crucial for maintaining the structural integrity throughout the charge–discharge process. Therefore, developing P2-type materials with higher Na content is a key objective. Such materials aim to retain more Na^+^ in the NaO_2_ slabs to suppress structural transitions while achieving or surpassing the capacities of materials with lower Na content (*x* = 2/3) (Figure 4a). To design high-Na-content P2-type materials, we found the cationic potential method has proven effective. For example, starting with the conventional low-Na-content P2-type Na_2/3_Ni_1/3_Mn_2/3_O_2_ (*x* = 2/3), which typically delivers a low initial charge capacity under a lower cutoff voltage and suffers from a P2 to O2 phase transition,^37^ the high-Na-content Na_5/6_Li_2/27_Ni_8/27_Mn_17/27_O_2_ composition was predicted by introducing Li^+^ to partially replace Mn^4+^/Ni^2+^. This adjustment increased the Na content to 5/6 per formula unit. The high-Na-content material demonstrated a significantly improved reversible capacity of ∼102.5 mAh g^–1^ within a voltage range of 2.0–4.0 V, exceeding the ∼82 mAh g^–1^ capacity of P2-type Na_2/3_Ni_1/3_Mn_2/3_O_2_ (Figure 4b). Additionally, it exhibited excellent long-term cycling stability, retaining 87% of its capacity after 1000 cycles. These results indicate that higher Na content promotes the oxidation of low-valent cations (e.g., Ni^2+^) to higher oxidation states at lower cutoff voltages during desodiation, enhancing the Na storage capacity. Simultaneously, it stabilizes the P2-type structure, generally promoting the Na-ion conductivity with enhanced stability. Our work has inspired future studies to design more efficient and durable P2-type materials, such as Na_0.80_Li_0.024_[Li_0.065_Ni_0.22_Mn_0.66_]­O_2_ ^38^ and Na_45/54_Mg_6/54_Ni_12/54_Mn_34/54_O_2_,^39^ providing a foundation for exploration of Na-ion battery cathodes. Moreover, possible dopant/substituting elements that may enable alternative high-Na-content P2-type materials are multivalent elements (e.g., Ca^2+^, Mg^2+^, Y^3+^, La^3+^), which, when located at the Na sites, will also promote the oxidation of TM ions and stabilize the structure upon (dis)­charging.

**Figure 4:**
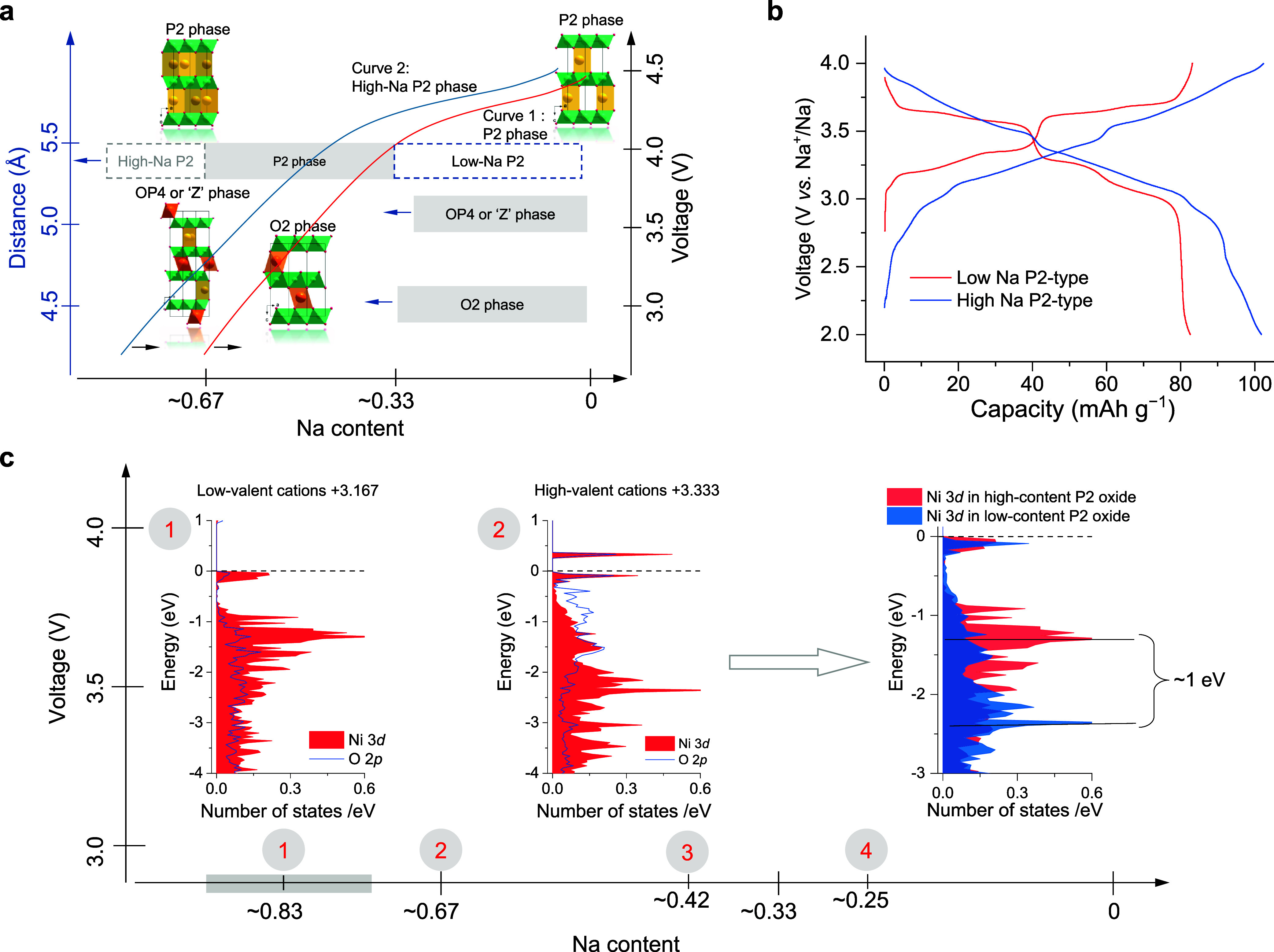
**Possible advantages of high-Na P2-type layered cathodes**. (a) Structural evolution mechanism of high-Na P2-type oxides upon charging. (b) Galvanostatic charge–discharge curves of low-Na Na_2/3_Ni_1/3_Mn_2/3_O_2_ and high-Na Na_45/54_Li_4/54_Ni_16/54_Mn_34/54_O_2_ electrodes in the voltage range 2.0–4.0 V. (c) Illustration of the electronic evolution mechanism in the Na_45/54_Li_4/54_Ni_16/54_Mn_34/54_O_2_ high Na-content P2-type material. The large amount of Na in high-Na P2-type materials reduces the average oxidation state of the TM ions below that of common low-Na-content P2 materials. The consequence is that the low-valent cations can be oxidized to their high state more easily. Reproduced from ref 3 Copyright 2020 American Chemical Society.

## 4 Low-Na-Content O3-Type Layered Cathodes

Typically, O3-type cathodes generally offer larger capacities than P2-type cathodes when operating at the same cutoff voltage.^2^ When charged to 4.0 V, P2-type Na_0.67_Ni_0.33_Mn_0.67_O_2_ retains a Na content of approximately 0.33 within its structure, resulting in a capacity of only about 84 mAh g^–1^. In contrast, the O3-type NaNi_0.5_Mn_0.5_O_2_ delivers a higher capacity, around 146 mAh g^–1^. However, attempting to fully exploit the theoretical capacity of P2-type cathodes by charging them to higher voltages (>4.15 V) introduces additional challenges, particularly harmful phase transitions. At these elevated voltages, the Na content is greatly reduced, leading to considerable structural instability and capacity loss.^3^ This instability prevents the realization of the large reversible capacity that P2-type cathodes could otherwise offer. In comparison, the O3-type cathodes undergo the O3-to-P3 phase transitions at lower voltages, during which a larger fraction of Na remains in the structure. Notably, the P3-type structure formed during the desodiation of the O3-type cathodes typically exhibits enhanced structural stability and facilitates faster Na-ion diffusion. As a result, when the capacity range of the P3-type structure is maximized, O3-type cathodes are expected to outperform P2-type cathodes in both capacity and structural integrity, presenting an opportunity for the development of improved O3-type layered cathodes.

Here, we obtained an O3-type Na_5/6_Li_2/27_Ni_8/27_Mn_11/27_Ti_6/27_O_2_ cathode with P3-dominated phase transition using the above high-Na P2-type Na_5/6_Li_2/27_Ni_8/27_Mn_17/27_O_2_ composition as a starting structural composition. We found that this P2-type structure, with nearly the maximum Na content, can be easily transformed into an O3-type structure through small compositional adjustments. By utilizing the cationic potential approach, partial substitution of redox-inactive Mn^4+^ with Ti^4+^ converts the P2-type structure into an O3-type structure, resulting in a shift that just crosses the dividing line between the P2- and O3-type phases (Figure 5a). This transition preserves a large *d*_(O–Na–O)_ interlayer distance, which promotes high Na^+^ diffusivity while realizing an O3-type structure. The resulting O3-type Na_5/6_Li_2/27_Ni_8/27_Mn_11/27_Ti_6/27_O_2_ has the largest *d*_(O–Na–O)_ interlayer distance among typical O3-type Na-ion layered cathodes, suggesting P2-like characteristics that facilitate enhanced Na^+^ diffusivity.

**Figure 5:**
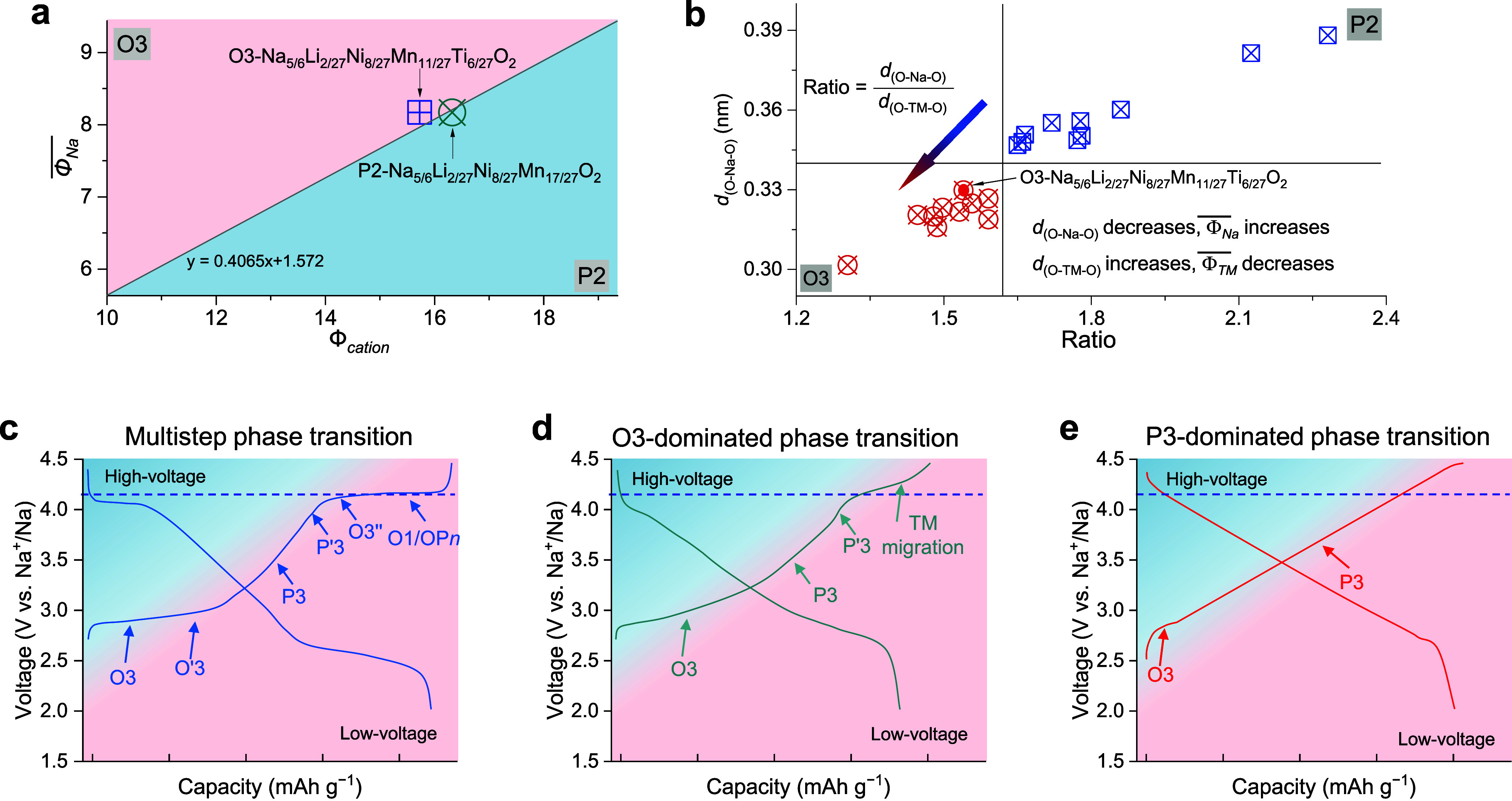
**Design of fast-charging O3-type layered electrode**. (a) Cationic phase map indicating the phase transition for P2-type Na_45/54_Li_4/54_Ni_16/54_Mn_34/54_O_2_ to the O3-type Na_5/6_Li_2/27_Ni_8/27_Mn_11/27_Ti_6/27_O_2_. (b) Plot of *d*_(O–Na–O)_ vs the ratio of the interlayer distances *d*_(O–Na–O)_ and *d*_(O–TM–O)_ for typical P2- and O3-type oxides and categorization of phase transitions in O3-type Na-ion 3d TM layered cathodes. (c) Multistep phase transition materials. (d) O3-dominated phase transition materials. (e) P3-dominated phase transition materials. Reproduced with permission from ref 2. Copyright 2024 the authors of ref 2, under exclusive license to Springer Nature.

This O3-type material exhibits high power density with high reversibility due to the P3-type dominant solid-solution reaction with open prismatic Na-ion diffusion pathways between the TMO_2_ slabs. Specifically, the P3-type phase tends to form at the beginning of the charge, and it continues to dominate, maintaining a solid-solution reaction as charging progresses. This is in contrast to materials that undergo multistep phase transitions, where the structural evolution starts with O3 and progresses through O′3, P3, P′3, O3″, and O1 phases during charging^40,45^ (Figure 5c). Additionally, compared to the case for the O3-dominated phase transition materials, which maintain the O3-type structure for a large portion of the charge capacity before transitioning to the P3-type structure (Figure 5d), the P3-dominated phase transition (Figure 5e) has distinct advantages in terms of structural stability and Na^+^ diffusivity. The short O3–P3 phase transition at low voltages, followed by the P3-type solid-solution transition at higher voltages, minimizes strain from volume changes and migration of TMs, thereby promoting long cycle life and rate capabilities.

In addition to the large *d*_(O–Na–O)_ interlayer distance, we found that the relatively low Na content (*x* = 5/6) in the O3-type structure also plays a crucial role. During charging, a small decrease in Na-ion composition, along with an increase in the Ni oxidation state, leads to reduced Na-ion shielding and strengthened TM–O interactions. These changes increase the *d*_(O–Na–O)_ distance and decrease the *d*_(O–TM–O)_ distance, facilitating the P3-dominated phase transition. This indicates that both the TM and the Na composition are pivotal factors in determining the phase transition behavior during charging, offering insights for the rational design of Na-ion layered oxide electrodes with improved performance.

## 5 Multicomponent High-Entropy O3-Type Layered Cathodes

In recent years, a significant focus has been placed on enhancing the Na-ion storage performance of layered materials by investigating the substitution or doping of TM sites in the Na_*x*_TMO_2_ system.^11,12^ This suggests that the development of multicomponent compounds could be a promising strategy for achieving property-oriented materials. Multicomponent materials, a concept that aligns with high-entropy materials,^41^ have been shown to exhibit a range of enhanced properties, including improved strength, good performance at both high and low temperatures, and superior energy storage capabilities. Multicomponent high-entropy oxides, such as the (MgCoNiCuZn)_1–*x*−*y*_Ga_*y*_A_*x*_O system,^42^ have demonstrated remarkable ion mobility. For instance, Li-ion mobility greater than 10^–3^ S cm^–1^ and Na-ion mobility around 5 × 10^–6^ S cm^–1^ have been observed. The underlying mechanism suggests that the incorporation of alkali metals into the material can lead to the creation of oxygen vacancies, which enhance the diffusion pathways and facilitate ion mobility. These improvements are attributed to the stabilization of the host lattice by the multicomponent composition, which helps maintain the rock-salt structure during cycling. Based on these findings, we have initially investigated the electrochemical properties for layered cathodes benefiting from the high-entropy chemistry.^4^

A multicomponent high-entropy cathode composed of nine metal ions with oxidation states ranging from bivalent to pentavalent, NaNi_0.12_Cu_0.12_Mg_0.12_Fe_0.15_Co_0.15_Mn_0.1_Ti_0.1_Sn_0.1_Sb_0.04_O_2_, has been identified as an O3-type structure using the cationic potential method. Within this composition, Ni^2+^, Cu^2+^, Fe^3+^, and Co^3+^ provide charge compensation for capacity, while Mg^2+^, Mn^4+^, Ti^4+^, Sn^4+^, and Sb^5+^ contribute to structural stabilization during the Na-ion (de)­intercalation process due to their different charges and sizes, as reflected by the ionic potentials. This composition was successfully prepared in the predicted O3-type structure based on the cationic potential method by a typical solid-state reaction. Transmission electron microscopy and energy-dispersive X-ray spectroscopy mapping revealed a uniform distribution of these elements, as depicted in Figure 6a. Compared to previously reported O3-type cathodes,^43–45^ our material exhibits a higher proportion of capacity (∼60%) stored in the plateau region corresponding to the O3 phase (Figure 6b,c). Additionally, this method demonstrates a highly reversible O3–P3 phase transition, which enhances long-term cycling stability and rate performance. Specifically, the cathode achieves ∼83% capacity retention after 500 cycles and retains ∼80% capacity at a current density of 5.0C. A mechanism indicates that the entropy stabilization effect within the host matrix likely facilitates the maintenance of the layered O3-type structure, as shown in Figure 6d, thereby supporting enhanced cycling stability and rate capability. This exploration of multicomponent high-entropy layered electrodes demonstrates a broader composition space for developing new materials with enhanced performance. These predictions could include the following: (1) the multiple components promote entropy stabilization within the host matrix, which helps accommodate changes in local interactions during ionic (de)­intercalation, leading to improved cycling stability and rate performance; (2) the diversity of components in the structure helps suppress phase transitions, increasing structural stability; (3) the multicomponent nature can also have an impact on the material’s morphology, resulting in more spherical shapes that influence performance; (4) by tuning the elements and their ratios, the electrochemical properties can be finely optimized, offering a wider compositional space to explore and develop new materials with enhanced performance. Following this work, many multicomponent O3-type cathodes obtained through controlling the Na contents and TM compositions have been reported, achieving further advancements in functionality, such as O3-type Na_0.9_Ni_0.25_Cu_0.05_Mg_0.05_Zn_0.05_Fe_0.05_Al_0.05_Mn_0.40_Ti_0.05_Sn_0.05_O_2_ ^46^ and O3-type Na_0.75_Ni_0.25_Cu_0.10_Fe_0.05_Mn_0.15_Ti_0.45_O_2_.^31^ Further work is recommended to integrate the cationic potential method for optimizing the ion composition in multicomponent high-entropy cathodes, thereby enhancing performance.

**Figure 6:**
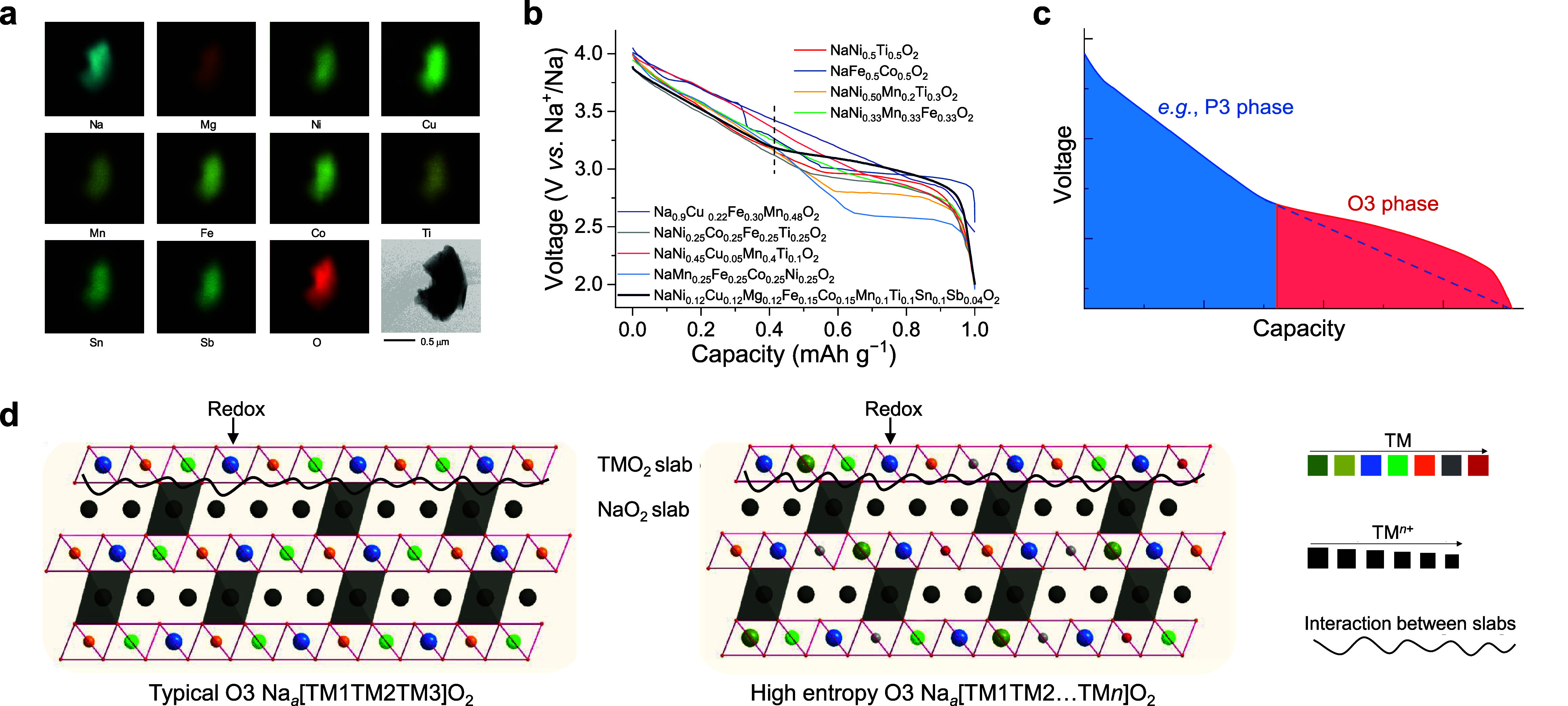
**Design of a high-entropy oxide electrode**. (a) Transmission electron microscopy image and energy-dispersive X-ray spectroscopy mappings for elements. (b) Discharge curves of the representative O3-type Na-ion cathodes. The vertical bar denotes the differentiation between the O3- and P3-type phases of a high-entropy cathode. (c) Illustration of typical discharge behavior for the O3-type cathodes, generally with the slope area occurring in the P3 phase and the plateau area in the O3 phase. (d) Possible mechanism of high-entropy composition in facilitating the layered O3-type structure. The conventional O3-type Na-ion cathodes feature three distinct types of TM elements, while the proposed high-entropy cathodes incorporate multiple TM elements. Two fingerprint spectra are employed to illustrate the types and oxidation states of elements occupying the TM sites in the O3-type cathodes. The colors indicate different TM element types, while the sizes reflect their oxidation states; larger sizes correspond to lower oxidation states. Wavy lines represent the local interactions between TM elements and Na ions within the TMO_2_ and NaO_2_ slabs. Reproduced with permission from ref 4. Copyright 2020 Wiley-VCH.

## 6 Outlook and Perspective

In this Account, we summarize our insights and progress in understanding and developing layered oxides for rechargeable battery technologies with a particular emphasis on Na-ion batteries due to their remarkable compositional diversity. This diversity introduces complex structural chemistry that critically influences layered stacking arrangements, ion conductivity, and redox activity, offering opportunities for the development of innovative functional material properties. By examining the structural and compositional variations in layered oxides, we propose the ionic potential as a quantitative tool to elucidate key interactions within the stacking structures. Indeed, the ionic radius is a crucial factor in designing inorganic crystalline materials, as it determines whether an ion can occupy specific sites within a crystal lattice. In battery materials, the ionic radius could impact the performance by influencing phase composition, structural stability, and ion diffusion kinetics. For instance, excessively large ions may distort the host lattice, compromising structural integrity during cycling, while smaller ions can enable faster diffusion but may also trigger undesirable side reactions or phase transitions. However, as research systems become more complex, relying solely on ionic radius proves insufficient for guiding element selection and material design.^47,48^ One key limitation is that many elements exhibit similar ionic sizes, making differentiation difficult.^49^ Moreover, the ionic radius does not account for cation–anion electrostatic interactions, which play a critical role in material behavior. To better capture these interactions, ionic charge must also be considered. This has led to the growing importance of ionic potential as a more comprehensive parameter for material design, providing a pathway for systematically designing representative crystal structures. Guided by this framework, we have investigated electrode materials aimed at achieving higher specific capacities (e.g., O3-type NaLi_1/3_Ti_1/6_Mn_1/2_O_2_ and P2-type Na_5/6_Li_5/18_Mn_13/18_O_2_) and enhanced rate capabilities (e.g., O3-type Na_5/6_Li_2/27_Ni_8/27_Mn_11/27_Ti_6/27_O_2_ and P2-type Na_45/54_Li_4/54_Ni_16/54_Mn_34/54_O_2_). While the performance of these materials may not yet be optimal, their meticulously controlled compositions and structures provide a valuable foundation for subsequent advancements. Furthermore, the exploration of multicomponent layered electrodes reveals a compositionally rich and largely unexplored space for developing superior material systems. This avenue holds promise for driving innovation in next-generation battery technologies.

To accelerate the practical application process, we discuss a potential principle which considers the nature of the chemical components and their roles in the materials. The common 3d TM elements used as redox centers in rechargeable batteries are Ti, V, Cr, Mn, Fe, Co, Ni, and Cu, and the general properties in layered electrodes are summarized in Figure 7a. Understanding the advantages and disadvantages of each TM element can aid in designing optimal cathodes for potential application, which should exhibit one or more of the following desired properties: large capacity, high potential, long-term cycling stability, high Coulombic efficiency, structural stability, and thermal stability. Additionally, a promising layered electrode could include several components, such as a structure former, charge compensator, structure stabilizer, and charge balancer (Figure 7b). The structure former should primarily consist of abundant TM elements such as Mn and Fe. The Mn^4+^ content can range from ∼0.4 to 0.6, provided that the discharge voltage remains above 2.0 V (preferably 2.5 V) to avoid Jahn–Teller distortion caused by the Mn^4+^/Mn^3+^ redox couple. Fe^3+^ can serve as a redox center, but its content should be limited to less than 0.2 to prevent structural instability from Fe^4+^ migration and excessive voltage polarization. The charge compensator is a key component of the electrodes, directly influencing the redox activity, battery voltage, capacity contribution, and dynamic performance. While its content is generally lower than that of the structure former, limiting its participation in redox reactions helps maintain host structure stability but impacts battery capacity. Mn^3+^, Fe^3+^, Ni^2+^/Ni^3+^, and Cu^2+^ are suitable charge compensators for cathodes. Ni^2+^ in particular is a promising multielectron charge compensator. Doping a proportion of Ni^2+^ into TMO_2_ slabs increases the capacity and supports high-voltage electrochemical reactions within the same voltage range, enabling a higher energy density. However, the Ni content should be limited to 0.15–0.35 per formula unit, contributing 30–60% of cathode capacity, considering its availability and cost. Structure stabilizers are also crucial for oxide cathodes, ensuring improved electrochemical performance and practical application. Common stabilizers include Mg^2+^, Cu^2+^, Ti^4+^, Al^3+^, and Li^+^, tailored to specific purposes such as suppressing phase transitions, enhancing air/water stability, mitigating Na^+^/vacancy or charge ordering, and maintaining structural integrity. All of the aforementioned factors can act as charge balancers. However, particular attention will be given to two components: Na vacancies (V_Na_) within NaO_2_ slabs and TM vacancies (V_TM_) within TMO_2_ slabs. For P2-type materials, the typical Na vacancy concentration is approximately 0.33, leading to a composition of [Na_0.67_(V_Na_)_0.33_]­TMO_2_. Reducing the Na-vacancy content allows for an increase in capacity due to a higher Na content. Therefore, we could prepare P2-type materials with a low Na vacancy content or O3-type materials with a high Na vacancy content, further providing additional opportunities to optimize material properties. By carefully selecting the elemental composition and leveraging ionic potential predictions to guide structural design, optimal cathode performance can be achieved through a systematic balance of these interdependent factors, tailored to meet application-specific requirements of advanced battery materials.

**Figure 7:**
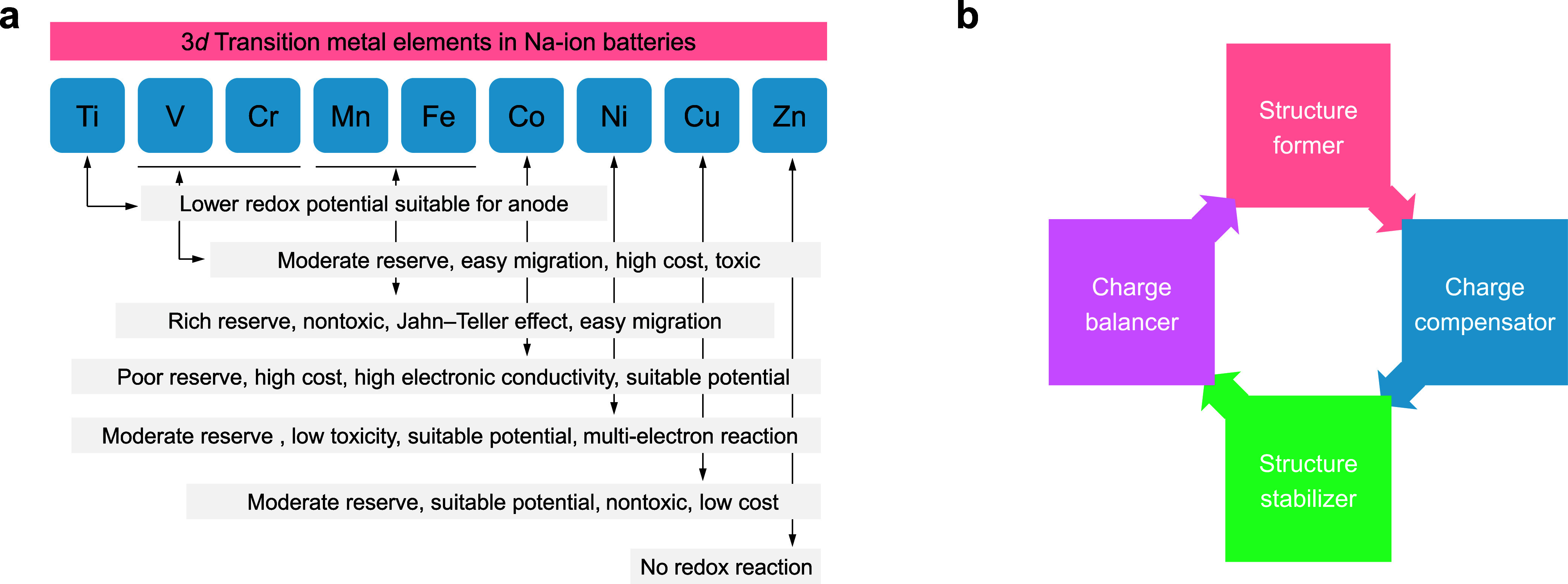
**Design principle of layered materials for batteries**. (a) Basic characteristics of 3d TM elements used for electrode materials. Reproduced with permission from ref 50 Copyright 2019 Tsinghua University Press and Springer Nature. (b) Components for the design of layered eletrodes, including the structure former, charge compensator, structure stabilizer, and charge balancer.
